# Near-Complete Whole-Genome Sequence of *Paenibacillus* sp. nov. Strain J5C2022, a Sucretolerant and Endospore-Forming Bacterium Isolated from Highly Concentrated Sugar Brine

**DOI:** 10.1128/mra.01055-22

**Published:** 2023-03-06

**Authors:** Siti Munirah Musa, Shing Wei Siew, Darren Dean Tay, Hajar Fauzan Ahmad

**Affiliations:** a Faculty of Industrial Sciences and Technology, Universiti Malaysia Pahang, Gambang, Pahang, Malaysia; University of Arizona

## Abstract

Here, we present a 7.62-Mbp genome sequence of *Paenibacillus* sp. nov. strain J5C2022, a Gram-positive facultatively anaerobic bacterium that was isolated from 4-month-old fruit pickle brine and sequenced using the Illumina platform.

## ANNOUNCEMENT

Gram-positive *Paenibacillus* spp., in the phylum *Firmicutes*, are endospore-forming bacteria that can switch between metabolically active or dormant states in response to stress or favorable conditions (1). They are facultatively anaerobic bacteria that grow optimally at temperatures between 28°C and 40°C under neutral pH conditions (2, 3). Strain J5C2022 was isolated from an overnight culture (on nutrient agar) of 4-month-old fruit pickle brine collected from a manufacturer in Penang, Malaysia. Briefly, 500 mL of brine was centrifuged at high speed, and the supernatant was discarded. The concentrated brine (100 μL) was spread onto a nutrient agar plate at 33°C for overnight cultivation. A single colony was picked randomly and identified using Sanger sequencing. Upon screening, strain J5C2022 was transferred to nutrient broth at 33°C for overnight cultivation to obtain a pure culture. Genomic DNA was extracted from the strain using the GenElute bacterial genomic DNA kit (Sigma-Aldrich Co., St. Louis, USA) as previously described (4). For the library preparation, 100 ng of DNA was measured and fragmented to 350 bp using a Bioruptor sonicator, which is compatible with the NEBNext Ultra II DNA library prep kit for Illumina (New England BioLabs Inc., Ipswich, USA). The sequencing was performed on the NovaSeq 6000 sequencing system (Illumina Inc., San Diego, USA) and produced 789,064,800 bp of paired-end reads (2 × 150 bp). The raw Illumina paired-end reads were trimmed using fastp v0.21 (5) to remove low-quality bases and Illumina adapter sequences. The trimmed reads were subsequently used for *de novo* assembly using Unicycler v0.4.8 (6). Contigs smaller than 500 bp, representing mostly sequencing artefacts, were removed, and the filtered assembly was used for subsequent analysis. Genome assembly statistics were generated using QUAST (7). The corresponding rRNA genes (5S, 16S, and 23S rRNA) were extracted using Barrnap (https://github.com/tseemann/barrnap) into a single FASTA file that was subjected to a BLAST search of the GTDB 16S rRNA database. The completeness of the genome was then analyzed using BUSCO v5 (https://gitlab.com/ezlab/busco), based on the Bacillales_odb10 lineage database (8). Unless otherwise noted, default parameters were used for all software tools. Strain J5C2022 has a total length of 7,616,378 bp, an *N*_50_ length of 137,040 bp, an average GC content of 51.6%, and 200× genome coverage. Genome annotation was carried out using the Rapid Annotations using Subsystems Technology (RAST) server (9) and the NCBI Prokaryotic Genome Annotation Pipeline (PGAP) with the GeneMarkS2 v1.14_1.25 method (10), resulting in 138 contigs with 6,340 coding sequences and 70 RNAs. A total of 856 genes were screened to be potentially involved in carbohydrate metabolism, covering the xylose and l-rhamnose utilization pathways. In addition to this strain’s ability to form endospores and its resistance to high salt and sugar conditions, its potential for use as a next-generation probiotic for application in the food industry is worth exploring.

### Data availability.

The complete genome sequence of *Paenibacillus* sp. nov. strain J5C2022 is available at NCBI under SRA accession number SRR19238564 and BioProject accession number PRJNA83215. The BioSample accession number is SAMN28409047. The GenBank accession number is JAOQJC000000000.1, with assembly accession number GCF_025567625.1.
